# Large-Volume Pneumoperitoneum in a Neonate: A Rare Case of Necrotizing Gastritis

**DOI:** 10.7759/cureus.103120

**Published:** 2026-02-06

**Authors:** Khaled Saed, Kayla L Haydon, Shree Patel, Gerald Gutwein, Logan Broome, Marthena Phan, Ameer Al-Hadidi, Leopoldo Malvezzi

**Affiliations:** 1 Surgery, Larkin Community Hospital, Miami, USA; 2 Surgery, Florida International University, Herbert Wertheim College of Medicine, Miami, USA; 3 Surgery, Hospital Corporation of America (HCA) Florida Westside Hospital, Plantation, USA; 4 Surgery, Cleveland Clinic Florida, Weston, USA; 5 Pediatric Surgery, Nicklaus Children's Hospital, Miami, USA

**Keywords:** gastric gangrene, idiopathic pneumoperitoneum, necrotizing gastritis, partial gastrectomy, spontaneous neonatal gastric perforation

## Abstract

Neonatal spontaneous gastric perforations are rare but are associated with high mortality rates. They usually occur within the first week of life, with a sudden onset of abdominal distension. Imaging often shows pneumoperitoneum. Gastric perforations require emergent surgical management.

We report a case of a two-day-old male with spontaneous patchy gastric gangrene and perforation of the greater curvature. The patient presented with abdominal distension after initiation of feeds. An X-ray of the abdomen revealed a large-volume pneumoperitoneum. The patient underwent emergent exploratory laparotomy, partial segmental gastrectomy, and primary closure in two layers. The patient started tolerating feeds on postoperative day 8.

Our case underscores the importance of considering neonatal spontaneous gastric perforation in the differential diagnosis of pneumoperitoneum. Large-volume pneumoperitoneum is a pathognomonic finding of necrotizing gastritis and gastric perforation in neonates and can lead to fatal respiratory decompensation. Early diagnosis and surgical intervention are imperative to improve prognosis.

## Introduction

Neonatal spontaneous gastric perforations are full-thickness defects of the neonatal stomach resulting in intraperitoneal leakage of acidic gastric contents and air, leading to peritonitis, potential respiratory failure, and sepsis. Spontaneous gastric perforations are reported in approximately one in 29,000 live births and are associated with high mortality rates of at least 60-70% [1]. Most cases present within the first week of life with acute abdominal distension and radiographic evidence of pneumoperitoneum, requiring emergent surgical intervention consisting of debridement and potential gastrectomy [2]. Prognosis after immediate surgical intervention is favorable [2].

The pathophysiology of neonatal gastric perforation remains incompletely understood. Proposed mechanisms include gastric ischemia related to perinatal hypoxia or stress; immaturity of the gastric muscularis propria, leading to focal wall weakness; increased intragastric pressure from early feeding or positive-pressure ventilation; and acid-mediated mucosal injury during the early neonatal period, when gastric acidity is relatively elevated [1-4]. These factors may act independently or synergistically to predispose the neonatal stomach to necrosis and perforation.

Importantly, spontaneous gastric perforation represents a distinct clinical entity from more common causes of neonatal pneumoperitoneum, such as necrotizing enterocolitis (NEC). NEC primarily affects the small and large intestines and typically presents later in the neonatal course after enteral feeding has been established [5]. In contrast, gastric perforation often presents earlier, may involve isolated gastric pathology, and is frequently associated with large-volume pneumoperitoneum without radiographic evidence of intestinal pneumatosis [3,6]. Due to its rarity and potentially life-threatening consequences, early recognition and differentiation from other causes of neonatal pneumoperitoneum are critical to improving outcomes.

## Case presentation

We report the case of a two-day-old male with neonatal gastric gangrene and perforation of the greater curvature. The patient was born at 36 weeks of gestational age via emergent cesarean section due to uterine cord prolapse resulting in intrauterine growth restriction. Maternal history was significant for hypertension and pre-eclampsia. The mother had been taking labetalol and received two doses of antenatal corticosteroids. All other prenatal serologies were negative, and there was no known tobacco exposure. Amniotic fluid was clear. The infant was delivered in vertex presentation, with a birth weight of 2.1 kg and Apgar scores of 8 at one and five minutes. He was admitted to the neonatal intensive care unit and initially required supplemental oxygen via continuous positive airway pressure but was subsequently weaned to room air.

**Figure 1:**
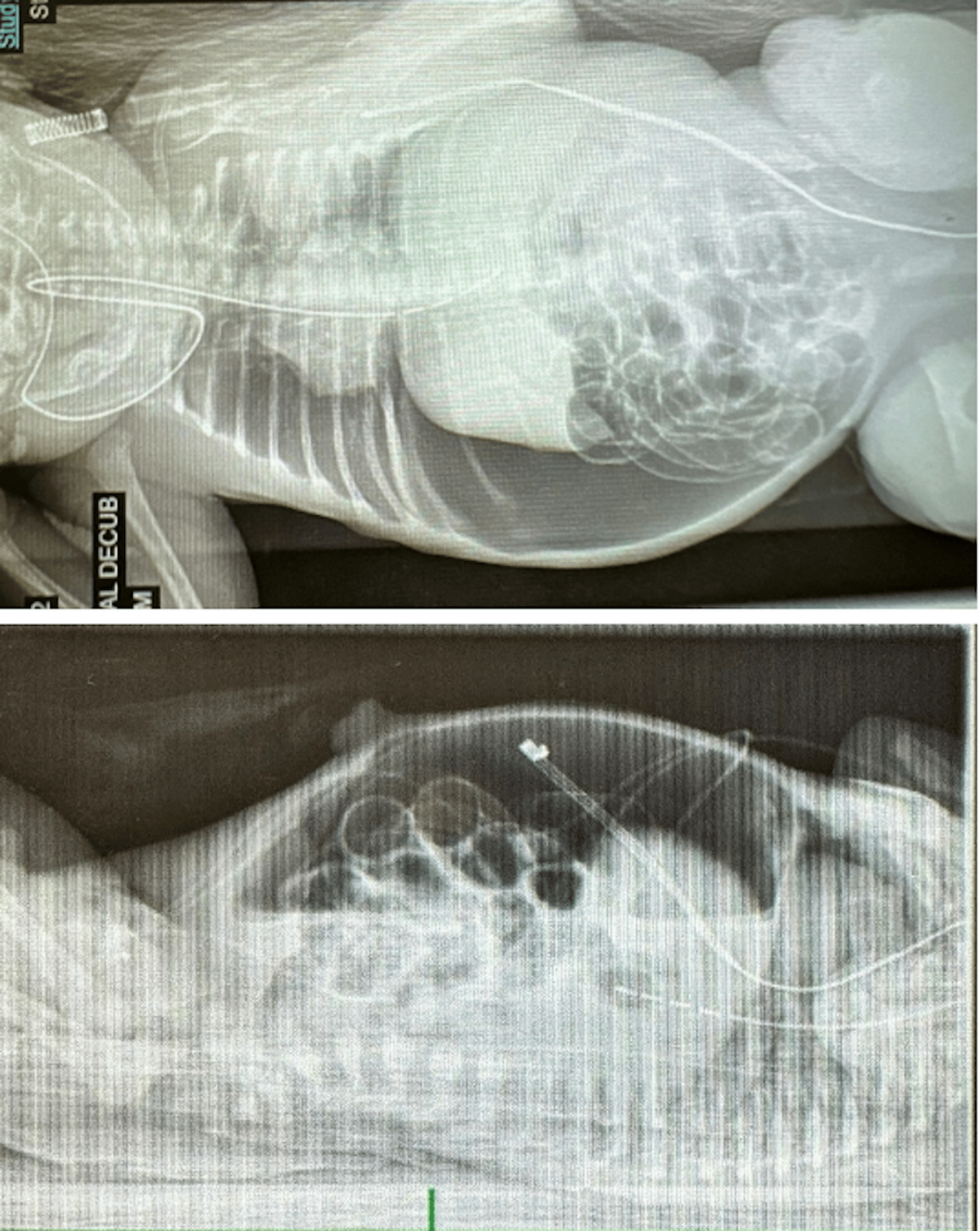
X-rays taken at day one of life indicating massive pneumoperitoneum Supine and decubitus abdominal radiographs obtained on day one of life demonstrate large-volume pneumoperitoneum with bowel loops outlined by free intraperitoneal air and displaced centrally. While intraluminal bowel gas is present, there is no radiographic evidence of pneumatosis intestinalis or portal venous gas.

After initiation of breastfeeding, the patient developed acute abdominal distension. Abdominal radiography demonstrated massive pneumoperitoneum (Figure 1). The initial differential diagnosis for neonatal pneumoperitoneum included NEC, intestinal perforation, iatrogenic gastric or bowel injury, and spontaneous gastric perforation. The absence of pneumatosis intestinalis or portal venous gas on imaging, the early presentation on day of life two, and the lack of preceding systemic illness made NEC less likely [5]. Given the large volume of free intraperitoneal air and concern for an upper gastrointestinal source, spontaneous gastric perforation was considered a leading diagnosis.

The patient was intubated for respiratory support, and a Replogle tube was placed for gastric decompression. Empiric broad-spectrum antibiotics, ampicillin and ceftazidime, were initiated to provide coverage for common neonatal pathogens. The patient was subsequently airlifted to our institution for definitive surgical management.

Upon admission, laboratory evaluation revealed metabolic acidosis on capillary blood gas analysis, with a pH of 7.32, partial pressure of carbon dioxide (pCO₂) of 32 mmHg, and bicarbonate (HCO₃⁻) level of 17.1 mmol/L. Capillary oxygen saturation was 84%. Serum lactic acid was elevated at 3.51 mmol/L, and the white blood cell count was 13.3 × 10⁹/L, without leukocytosis. Given concern for intra-abdominal contamination and potential sepsis, antibiotic therapy was broadened to include meropenem and vancomycin for expanded coverage of gram-negative, anaerobic, and gram-positive organisms.

**Figure 2:**
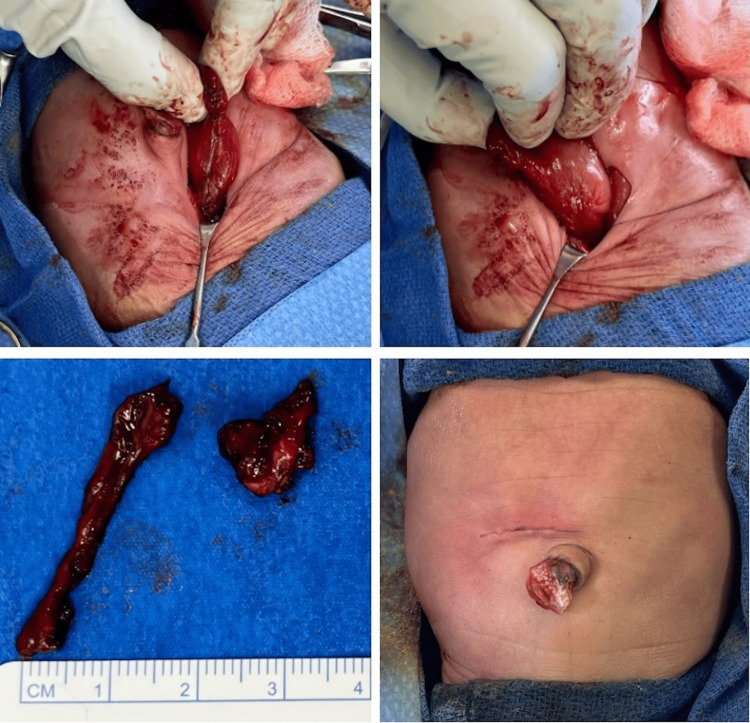
Partial segmental gastrectomy and primary closure in two layers

The patient underwent emergent exploratory laparotomy, which revealed patchy gastric gangrene and necrotic tissue along the greater curvature of the stomach without evidence of small or large bowel ischemia or perforation. Partial segmental gastrectomy of the necrotic tissue was performed, followed by primary closure in two layers (Figure 2).

Postoperatively, the patient was managed with total parenteral nutrition, intravenous fluids, and a 14-day course of piperacillin-tazobactam to provide continued broad-spectrum antimicrobial coverage given the extent of gastric necrosis and risk of intra-abdominal infection. A Replogle tube was maintained to low intermittent suction with bilious output. The abdomen remained soft and nondistended on serial examinations. Bowel function returned on postoperative day two. On postoperative day five, an upper gastrointestinal contrast study demonstrated focal contrast pooling along the proximal gastric suture line without evidence of extraluminal leak. Enteral feeds were initiated on postoperative day eight and were gradually advanced without complication.

## Discussion

Although the first case of spontaneous gastric perforation was reported in 1825, the etiology of neonatal gastric perforations remains incompletely understood [3]. Several mechanisms have been proposed, including gastric ischemia related to perinatal hypoxia or stress, immaturity of the gastric muscularis propria resulting in impaired motility and focal wall weakness, increased intragastric pressure from early feeding or positive-pressure respiratory support, and acid-mediated mucosal injury during the early neonatal period when gastric acidity is relatively elevated [1,2,4,6,7]. These factors may act independently or synergistically to predispose the neonatal stomach to necrosis and perforation.

The most commonly reported risk factors for neonatal gastric perforation include preterm birth and low birth weight. Prematurity is associated with reduced maturity of gastric smooth muscle cells, resulting in impaired gastric motility and increased intragastric pressure [1]. Additional gestational and intrapartum risk factors described in the literature include premature rupture of membranes, pregnancy toxemia, breech presentation, maternal diabetes, placenta previa, amniotic infection, and cesarean delivery [1]. Medical and postnatal factors such as ventilator use or other respiratory resuscitative measures, trauma related to endotracheal tube placement, and neonatal steroid exposure have also been implicated [1,6,7]. It has further been hypothesized that gastric perforations preferentially occur within the first week of life due to peak gastric acid secretion during this period [2]. Our case is consistent with these observations, as the patient was premature, low birth weight, delivered via cesarean section due to uterine cord prolapse, and required continuous positive airway pressure for oxygen supplementation. The perforation was identified on day of life two.

Clinically, neonatal gastric perforation most often presents with acute abdominal distension, feeding intolerance, respiratory distress, and, in severe cases, hemodynamic instability or shock [4]. Radiographic evaluation typically demonstrates pneumoperitoneum, which may be large-volume in early neonates [2-4]. In our patient, abdominal distension and massive pneumoperitoneum on plain radiography were the predominant presenting features. Large-volume pneumoperitoneum in early neonates is considered highly suggestive of gastric perforation and can rapidly lead to respiratory compromise due to diaphragmatic elevation and reduced lung compliance [2,6].

Differentiating spontaneous gastric perforation from more common causes of neonatal pneumoperitoneum, particularly NEC, is critical, as management and prognosis differ substantially [5]. NEC is an inflammatory disease that primarily affects the small and large intestines and typically presents later in the neonatal course after enteral feeding has been established [5]. Characteristic radiographic findings of NEC include pneumatosis intestinalis, portal venous gas, and fixed dilated bowel loops [5]. In contrast, spontaneous gastric perforation often presents within the first few days of life, may involve isolated gastric pathology, and is frequently associated with large-volume pneumoperitoneum without radiographic evidence of pneumatosis intestinalis [3,4,6]. In this case, the early presentation, the absence of pneumatosis intestinalis or portal venous gas on imaging, and intraoperative findings of isolated gastric gangrene without intestinal involvement strongly argue against NEC and support a diagnosis of primary gastric necrosis and perforation.

Prior to surgical intervention, suspicion of gastric perforation warrants prompt gastric decompression, most commonly via placement of a Replogle tube. In cases of severe respiratory compromise, emergent peritoneal decompression via needle or peritoneostomy puncture has been described as a life-saving temporizing measure to relieve intraperitoneal pressure and improve ventilation [6,7]. Definitive management requires emergent exploratory laparotomy.

Surgical repair of neonatal gastric gangrene and perforation typically consists of debridement and primary gastric closure, with partial or total gastrectomy reserved for cases with extensive necrosis [3]. Published case reports and small case series describe similar presentations characterized by early-onset abdominal distension, massive pneumoperitoneum, and gastric perforation, most commonly involving the greater curvature [2-4,6,7]. Surgical management has ranged from primary closure to partial or total gastrectomy, depending on the extent of tissue involvement, with outcomes determined mainly by the timeliness of diagnosis and intervention [2,3,7]. Consistent with these reports, our patient required partial segmental gastrectomy due to focal gastric gangrene and demonstrated a favorable postoperative course with return of bowel function and successful advancement of enteral feeds.

Overall, this case highlights the importance of maintaining a high index of suspicion for spontaneous gastric perforation in early neonates presenting with large-volume pneumoperitoneum. Prompt recognition, careful exclusion of alternative etiologies such as NEC, and timely surgical intervention remain essential to preventing respiratory decompensation and improving outcomes in this rare but life-threatening condition.

## Conclusions

This case reinforces the importance of considering spontaneous gastric perforation and necrotizing gastritis in the differential diagnosis of neonatal pneumoperitoneum, particularly when presentation occurs early in life and imaging demonstrates large-volume free intraperitoneal air without evidence of intestinal pneumatosis. Prompt diagnosis and early surgical intervention are critical to preventing respiratory decompensation and improving prognosis in affected neonates.
